# OX40 Stimulation Enhances Protective Immune Responses Induced After Vaccination With Attenuated Malaria Parasites

**DOI:** 10.3389/fcimb.2018.00247

**Published:** 2018-07-19

**Authors:** Ahmad Syibli Othman, Blandine M. Franke-Fayard, Takashi Imai, Esmé T. I. van der Gracht, Anke Redeker, Ahmed M. Salman, Catherin Marin-Mogollon, Jai Ramesar, Séverine Chevalley-Maurel, Chris J. Janse, Ramon Arens, Shahid M. Khan

**Affiliations:** ^1^Leiden Malaria Research Group, Parasitology, Leiden University Medical Center, Leiden, Netherlands; ^2^Faculty of Health Sciences, Universiti Sultan Zainal Abidin, Terengganu, Malaysia; ^3^Department of Immunohematology and Blood Transfusion, Leiden University Medical Center, Leiden, Netherlands; ^4^The Jenner Institute, University of Oxford, Oxford, United Kingdom

**Keywords:** malaria, *Plasmodium*, GAP, immunization, OX40, T cells, vaccine

## Abstract

Protection against a malaria infection can be achieved by immunization with live-attenuated *Plasmodium* sporozoites and while the precise mechanisms of protection remain unknown, T cell responses are thought to be critical in the elimination of infected liver cells. In cancer immunotherapies, agonistic antibodies that target T cell surface proteins, such as CD27, OX40 (CD134), and 4-1BB (CD137), have been used to enhance T cell function by increasing co-stimulation. In this study, we have analyzed the effect of agonistic OX40 monoclonal antibody treatment on protective immunity induced in mice immunized with genetically attenuated parasites (GAPs). OX40 stimulation enhanced protective immunity after vaccination as shown by an increase in the number of protected mice and delay to blood-stage infection after challenge with wild-type sporozoites. Consistent with the enhanced protective immunity enforced OX40 stimulation resulted in an increased expansion of antigen-experienced effector (CD11a^hi^CD44^hi^) CD8^+^ and CD4^+^ T cells in the liver and spleen and also increased IFN-γ and TNF producing CD4^+^ T cells in the liver and spleen. In addition, GAP immunization plus α-OX40 treatment significantly increased sporozoite-specific IgG responses. Thus, we demonstrate that targeting T cell costimulatory receptors can improve sporozoite-based vaccine efficacy.

## Introduction

Malaria remains a major threat to the lives of more than 3 billion people world-wide and there remains a pressing but unmet need for an effective vaccine, which can provide sustained protection against either infection or disease. Despite three decades of clinical testing different (recombinant) sub-unit vaccines, only modest protection has been reported so far (Hoffman et al., 2015; Tinto et al., 2015; White et al., 2015; Mahmoudi and Keshavarz, 2017) and this has renewed an interest in whole parasite-based vaccine approaches (Pinzon-Charry and Good, 2008; Hollingdale and Sedegah, 2017). It was first shown in rodent models of malaria that complete protection against infection can be obtained by vaccination using live attenuated sporozoites (Nussenzweig et al., 1967, 1969). Sterile protection against a malaria infection was also demonstrated in humans after immunization with *Plasmodium falciparum* sporozoites, either attenuated by radiation or administered under chemoprophylaxis (Hoffman et al., 2002; Roestenberg et al., 2009; Seder et al., 2013). A prerequisite for induction of protective immunity using sporozoite-based vaccines is that sporozoites retain their capacity to invade liver cells after their administration. The most advanced live-attenuated vaccine is based on radiation-attenuated sporozoites (PfSPZ-Vaccine), which is currently being evaluated both in the clinic and in field trials (Richie et al., 2015; Sissoko et al., 2017). In rodent models, immunization with sporozoites of genetically-attenuated parasites (GAP) can induce similar or even better levels of protective immunity compared to irradiated sporozoites (Irr-Spz) (Butler et al., 2011; Othman et al., 2017). Rodent GAP studies have been critical in the creation of two *P. falciparum* GAP-based vaccines that are currently undergoing clinical evaluation (Khan et al., 2012; Mikolajczak et al., 2014; van Schaijk et al., 2014).

A number of studies from both the clinic and the field have shown that Irr-Spz can generate strong protective immunity in humans (Ishizuka et al., 2016; Lyke et al., 2017; Sissoko et al., 2017). However, in order to achieve high level protective immunity multiple immunizations with high doses of attenuated sporozoites are required (Seder et al., 2013; Sissoko et al., 2017). The high numbers of sporozoites required for vaccination increases the costs of sporozoite-based vaccines and complicates the production and application of such vaccines for mass administration in malaria-endemic countries. The major challenge is to produce a highly immunogenic live-attenuated vaccine, which requires the fewest attenuated sporozoites per dose and the fewest doses to induce sustained sterile protection against a malaria infection.

While the precise mechanisms of protection mediated by immunization with attenuated sporozoites remain unknown, T cells appear to be critical for protection and in particular CD8^+^ T cells are thought to play a major role in eliminating infected hepatocytes. Early rodent studies using Irr-Spz have demonstrated a vital role for CD8^+^ T cells (Schofield et al., 1987; Weiss et al., 1988). Recent mechanistic investigations into protective immune responses induced by immunization with attenuated sporozoites have demonstrated diverse and robust immune responses that encompasses both CD8^+^ and CD4^+^ T cells, as well as a significant contribution from antibodies (Doll and Harty, 2014; Van Braeckel-Budimir et al., 2016). Nonetheless, CD8^+^ T cells are considered to be the main effector cells in eliciting protection after sporozoites immunization (Silvie et al., 2017).

Recently, cancer immunotherapies have employed antibodies that target proteins on the surface of T cells, as treatment with these antibodies have been shown to restore, expand and enhance the function of tumor-reactive T cells. The antagonistic antibodies targeting CTLA-4 and PD-1 have been used to block inhibitory signals to T cells (Curran et al., 2010; Wolchok et al., 2013), while agonistic antibodies targeting CD27, OX40, and 4-1BB on CD4^+^ and CD8^+^ T cells have been used to increase costimulatory signals (Croft, 2003; Dawicki et al., 2004; Melero et al., 2007). These immunostimulatory antibodies have been shown to improve the control of tumors and this was associated with an increase in tumor-specific T cell function (Schaer et al., 2014). In this study, we have analyzed the effect of agonistic OX40 monoclonal antibody (OX40 mAb) treatment on protective immunity induced in mice by immunization with GAP sporozoites. We immunized BALB/c mice using sporozoites of a *P. yoelii* GAP, an established rodent model to evaluate GAP vaccination (Butler et al., 2011). We found that OX40 mAb (α-OX40) treatment enhanced protective immunity, which was correlated with an expansion effector CD4^+^ and CD8^+^ T cell subsets, in both the liver and the spleen. In addition α-OX40 treatment induced the production of effector cytokine-producing T cells in the liver and spleen. Our results indicate that targeting costimulatory receptors on T cells can be used to improve sporozoite-based vaccine potency and in turn could be used to improve GAP vaccine implementation by reducing the numbers of sporozoites required to induce protective immunity.

## Materials and methods

### Experimental animals and parasites

Female BALB/cByJ mice (6–7 weeks; Charles River, NL and Harlan, Bicester, UK) were used. All animal experiments of this study were approved by the Animal Experiments Committee of the Leiden University Medical Center (DEC 13132 and 14307). The Dutch Experiments on Animal Act is established under European guidelines (EU directive no. 86/609/EEC regarding the Protection of Animals used for Experimental and Other Scientific Purposes). All experiments were performed in accordance with relevant guidelines and regulations. Two *P. yoelii* (*Py*) lines were used: (i) the reference “wild type” *Py*17XNL parasite line 1971cl1 (PyWT; PyGFP-luc_con_; line RMgm-689; www.pberghei.eu (Lin et al., 2011); which contains the fusion gene *gfp-luc* gene under control of the constitutive *eef1*α promoter integrated into the silent *230p* gene locus (PY17X_0306600) and does not contain a drug-selectable marker and (ii) the “genetically attenuated parasite” *Py*17XNL mutant that lacks the gene *fabb/f* (3-oxoacyl-acyl-carrier protein synthase; PY17X_1126500). This mutant (ΔPyFabBF-GFP-Luc_con_; *Py*Δ*fabb/f* ; mutant RMgm-4109; www.pberghei.eu) was generated in the reference line 1971cl1 (Haeberlein et al., 2017) by standard methods of transfection using a DNA construct that targets the *fabb/f* gene containing *hdhfr/fcu* selectable marker cassette by double cross-over integration.

### Mosquito infection, analysis of oocysts and preparation and injection of sporozoites

Sporozoites were obtained by manual dissection of the salivary glands of infected female *Anopheles stephensi* mosquitoes 14 days after feeding on infected mice. Mosquitoes were kept at a temperature of 24.5°C and 80% humidity. Salivary glands were collected in RPMI medium, homogenized and filtered (40 μm Falcon, Corning, Amsterdam, NL). The free sporozoites were counted in a Bürker counting chamber using phase-contrast microscopy. For intravenous (IV) administration sporozoites were suspended in RPMI medium and per mouse 200 μl was injected into the tail vein. Oocyst numbers in dissected midguts from infected mosquitoes were established 8 days after feeding using light-microscopy.

### Determination of parasite liver load by real time *in vivo* imaging

Parasite liver loads in live mice after immunization and after challenge were quantified by real time *in vivo* imaging as previously described (Annoura et al., 2013). Liver stages were visualized and liver loads quantified by measuring luciferase activity of parasites in whole bodies of mice at 44 h after injection of sporozoites using the IVIS Lumina II Imaging System (Perkin Elmer Life Sciences, Waltham, USA). During measurements mice were anesthetized using the isofluorane-anesthesia system (XGI-8, Caliper Life Sciences, Hopkinton, USA). D-luciferin was dissolved in PBS (100 mg/kg; Caliper Life Sciences, USA) and injected subcutaneously in the neck. Measurements were performed within 8 min after the injection of D-luciferin. Quantitative analysis of bioluminescence of whole bodies was performed by measuring the luminescence signal intensity using the ROI (region of interest) settings of the Living Image® 4.4 software.

### Immunization protocol and determination of prepatent period after challenge

For the immunization experiments mice were immunized using isolated *Py*Δ*fabb/f* sporozoites according to the immunization protocols described in the Results section. Blood of immunized mice was analyzed for possible breakthrough blood infections by Giemsa-stained blood smears 1 day before challenge with PyWT sporozoites. Immunized mice and naïve controls were challenged 14 days after the last immunization with 3000 (i.e., 3 × 10^3^) PyWT sporozoites. Challenged mice were monitored for blood-stage infections by Giemsa-stained blood smears made at day 4 to 14 after challenge. The prepatent period (measured in days after sporozoites challenge) is defined as the day when a blood stage infection with a parasitemia of 0.5–2% is observed (van Schaijk et al., 2014). Organs (and serum) used for immunological analysis were collected from the mice at day 7 after immunization or at 7 days after challenge.

### OX40 monoclonal antibody (mAb) treatment

Mice were treated with 200 μg of OX40 mAb (clone RM134L; Bio X Cell, West Lebanon, NH, United States) in 200 μl PBS and administrated by intraperitoneal injection (IP) either at day 0 or one day after prime or boost immunization.

### Treatment with ARTC2-blocking nanobodies

Immunized mice and naïve controls were treated with 50 μg ARTC2-blocking nanobodies (Biolegend) in 200 μl PBS administered by IP injection 30 min before sacrificing mice for collection of the organs for the immunological assays.

### Liver perfusion and purification of liver and spleen cells

Mice were perfused under anesthesia by intracardiac injection of 20 ml PBS (B. Braun, Oss, NL). Perfused livers were minced in small pieces and digested for 30 min at 37°C in Dulbecco's Modified Eagle Medium (Thermo Fisher Scientific, Breda, NL) containing 250 U/ml collagenase and 20 μg/ml DNase. Hepatic leukocytes was obtained by passing the digested tissue through a 70 μM cell-strainer (BD Biosciences. San Diego, CA) and Percoll gradient. For spleens, splenocytes were harvested by mincing the tissue through a 70 μm cell strainer.

### Cell surface staining, intracellular staining, and flow cytometry

For cell surface staining, hepatic leukocytes and splenocytes were resuspended in staining buffer (PBS, 2% FCS. 0.05% sodium azide) and incubated with fluorescent conjugated Abs for 30 min at 4°C. For intracellular cytokine staining, hepatic leukocytes and splenocytes were re-stimulated *in vitro* with medium containing 5 × 10^4^ PyWT sporozoites for 24 h in 96-well flat-bottom plates (1.5 × 10^6^ hepatic leukocytes and splenocytes per well) as described (Arens et al., 2011). In order to improve re-stimulation and to increase the number of antigen-presenting cells, 1.5 × 10^5^ of splenocytes were added at the start of the cultures to all wells. Twenty hours after incubation 1 μg/ml brefeldin A (Golgiplug; BD Pharmingen) was added to all wells. After re-stimulation, cells were transferred to U-bottom 96-well plates, and the cell surface stained with fluorescent conjugated Abs at 4°C for 30 min in staining buffer. After washing, cells were fixed with 0.5% paraformaldehyde at 4°C for 30 min, followed by intracellular staining for cytokines at 4°C for 30 min in Perm/Wash buffer (BD Biosciences). After washing and resuspending in staining buffer, cells were acquired using a BD LSRII flow cytometer and data were analyzed using FlowJo software (Tree Star). Fluorochrome-conjugated mAbs specific for CD3, CD4, CD8, CD44, CD11a, KLRG1, CD134, IFN-γ, IL-2, and TNF were purchased from BD Biosciences or eBioscience (San Diego, CA).

### Elisa

Enzyme-linked immunosorbent assay (ELISA) plates (Corning, Inc.) were coated overnight at 4°C by adding 1 × 10^4^ *Py*Δ*fabb/f* sporozoite lysate diluted in 100 μl NaHCO_3_ buffer (pH 9,6) per well. Plates were washed three times with PBS-T (0.05% Tween 20 in 1 × PBS) prior to blocking for 2 h in blocking buffer (1% BSA in PBS-T). Next, sera was diluted in blocking buffer at 1:100 for sporozoite lysate per well. Plates were incubated for 3 h at room temperature before washing as described above. Next, 100 μl of a 1:5000 dilution of horseradish peroxidase (HRP) conjugated anti-mouse IgG (Jackson Immuno-Research) was added and incubated for an additional 1 h at room temperature. Finally, plates were washed again and 100 μl of TMB Substrate solution (Thermo Scientific) was added for 5 min. The reaction was stopped by addition of 50 μl of 0.5 N sulfuric acid prior to measurement of absorbance at 450 nm using a Multiskan FC (Thermo Scientific) microplate reader.

### Statistics

All data are calculated using the GraphPad Prism software package 5.04 (GraphPad Software, Inc). For ELISA, cell surface and intracellular staining analysis, statistical analysis was performed using the unpaired Student's *t*-test. For the survival analysis, statistical analyzes to determine differences in protection after challenge were performed using a Kaplan–Meier survival plot, and survival curves were compared using the log-rank (Mantel-Cox) test. Survival was considered as the complete absence of parasites in blood. The significance threshold were 0.05 in all analysis.

## Results

### Establishing a gap Spz-BALB/C immunization-challenge protocol to investigate strategies to improve gap immunization

The *P. yoelii*-BALB/c parasite-mouse combination is a well-established model used to analyze vaccines that target sporozoites or liver-stage parasites. To analyze protective immunity induced after GAP immunization, we used sporozoites of *P. yoelli* Δ*fabb/f* GAP parasites. The *Py*Δ*fabb/f* parasites (GAP) lacks the *fabb/f* gene (PY17X_1126500) and arrest late into liver stage development (Vaughan et al., 2009; Haeberlein et al., 2017). This GAP produces oocysts and salivary gland sporozoites comparable to the wild-type parent *P. yoelii* 17XNL PyGFP-luc_con_ line (PyWT) (Figure S1A). Both GAP and PyWT sporozoites express the fusion protein GFP-Luciferase under control of the constitutive *eef1a* promoter, permitting the determination of parasite liver loads in live mice by real time bioluminescence imaging (Haeberlein et al., 2017). The GAP sporozoites exhibit levels of *in vivo* liver infection that are comparable to PyWT sporozoites (Figure S1B), however, these GAP sporozoites are unable to initiate a blood infection (Table S1).

**Figure 1:**
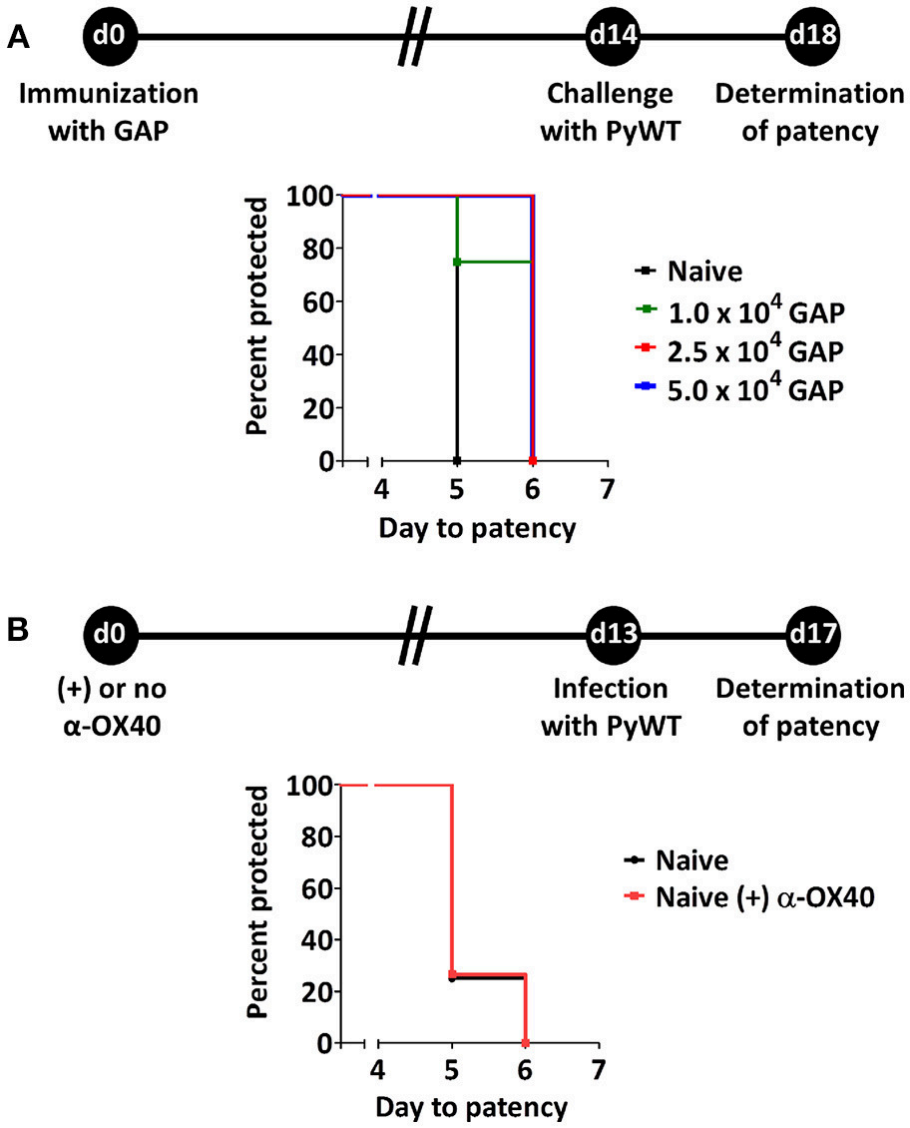
Suboptimal protection after GAP immunization and effect of α-OX40 treatment on prepatent period after infecting mice with wild type (PyWT) sporozoites. **(A)** Protection assays performed in groups of BALB/c mice (*n* = 5 per group) immunized with as single dose of 1, 2.5, or 5 × 10^4^ GAP sporozoites and challenged 14 days later with 3 × 10^3^ PyWT sporozoites. Challenged mice were monitored for blood-stage infections. The Kaplan-Meier curves illustrate the prepatent period (day at which a parasitemia of 0.5–2% is observed). Immunization with 2.5 and 5 × 10^4^ GAP sporozoites resulted in a significant longer prepatent period compared to control, non-immunized mice [Log-Rank (Mantel-Cox) test]. **(B)** Effect of α-OX40 treatment on parasite development in liver and blood. BALB/c mice were treated with α-OX40 on day 0 and infected with 3.0 × 10^3^ PyWT sporozoites 13 days later. Infected mice were monitored for blood-stage infections. The Kaplan-Meier curves illustrate that there was no significant differences were observed in prepatent period between α-OX40 treated mice (*n* = 15) and control, non-treated mice (*n* = 8) in two experiments.

Protective immunity in immunized mice after challenge with WT sporozoites is defined either by the number of mice that are completely protected from infection or by the delay in time taken to establish a blood stage infection, i.e., the prepatent period (time-to-event analysis) (O'Meara et al., 2007). In this study the prepatent period is defined as the number of days to reach a 0.5-2% parasitemia after PyWT sporozoites challenge as previously described (van Schaijk et al., 2014). Previously we had established that a primary immunization followed by boost immunization with 1 × 10^4^ GAP sporozoites induced sterile protection in more than 90% of BALB/c mice against challenge with 1 × 10^4^ PyWT sporozoites (Haeberlein et al., 2017). To examine putative enhancing protective immunity of treatment with adjuvants/immunomodulatory molecules we attempted to identify a “sub-saturating” immunization regiment by immunizing mice with only a single dose of GAP parasites. Mice were immunized with either 1, 2.5, or 5 × 10^4^ GAP sporozoites and then challenged 14 days later with 3 × 10^3^ PyWT sporozoites (Figure 1A). A single immunization with all three doses resulted in none of the mice being completely protected. We observed a maximum of 1 day delay in prepatent period in immunized mice compared to naïve mice. Since the blood stage multiplication rate is 10 × per 24 h, a 1 day delay in the prepatent period of blood stage infection represents 90% reduction in the infection in the liver (Janse et al., 2006). The immunization with 2.5 and 5 × 10^4^ GAP sporozoites resulted in a significance longer prepatent period (“survival”; *p* = 0.014 and *p* = 0.025, respectively) compared to naïve mice after challenge with PyWT sporozoites. Since we did not observe a major difference between the dose of 2.5 and 5 × 10^4^, we choose the protocol of 2.5 × 10^4^ GAP immunization followed by 3 × 10^3^ PyWT sporozoites challenge, to analyze the effect of α-OX40 treatment on protective immunity.

In addition, we performed an experiment to analyze the possible effect of treatment with α-OX40 in naive mice on liver and/or blood stage infection. Naïve mice treated with 200 μg of α-OX40 in 200 μl PBS by intraperitoneal injection (IP) and non-treated mice were challenged with PyWT sporozoites 14 days after treatment. No differences in prepatent period were observed between treated and untreated mice (Figure 1B), indicating that α-OX40 treatment has no effect on growth/multiplication of PyWT parasites in both the liver and blood in non-immunized mice.

### α-OX40 treatment increases the protective immunity in mice immunized with a single gap immunization

**Figure 2:**
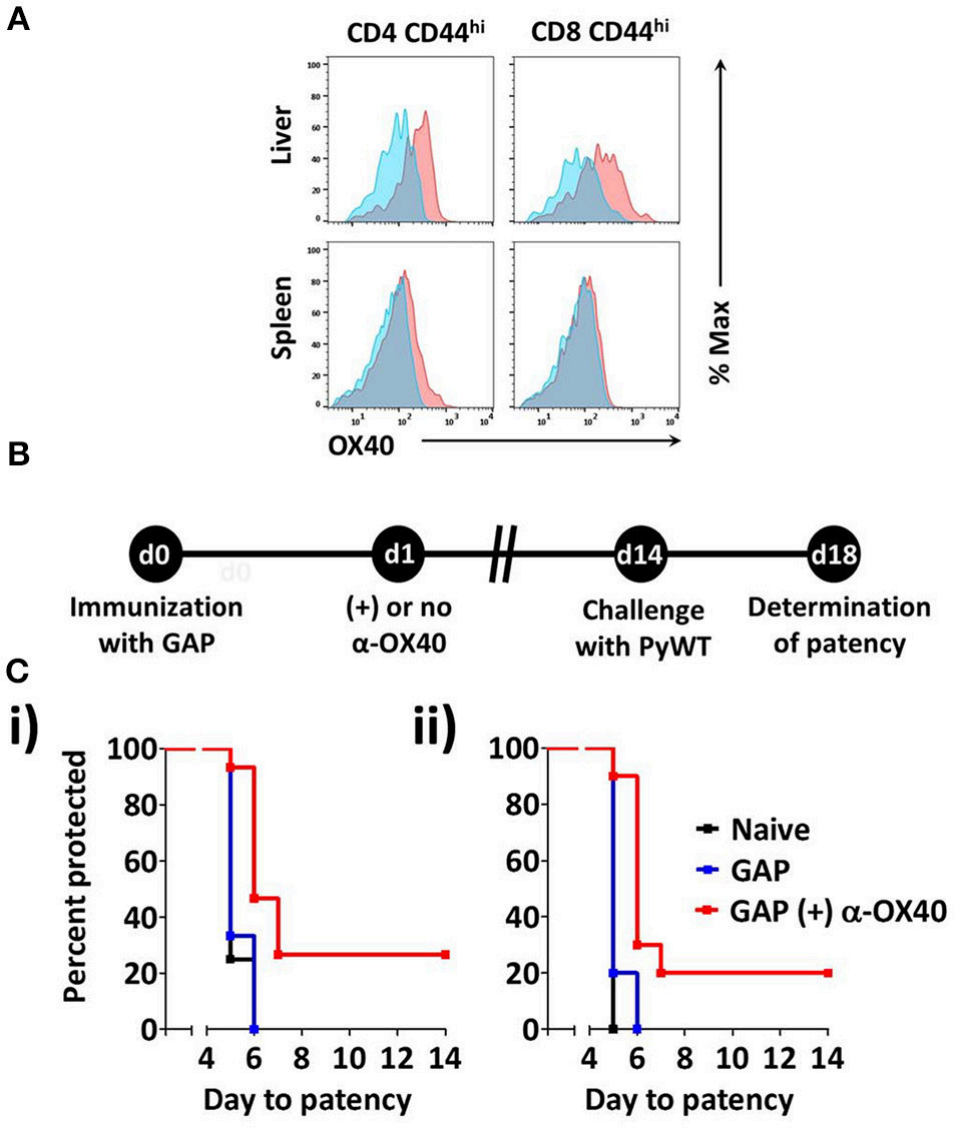
α-OX40 treatment increases the protective immunity in mice that received a single GAP immunization. **(A)** OX40 expression on activated CD44^hi^ CD4^+^ and CD44^hi^ CD8^+^ T cells in the spleen and liver at day 3 after immunization. Flow cytometric histograms indicate OX40 expression (red) and fluorescence minus-one (FMO) controls (blue). **(B)** The time line shows immunization of BALB/c mice with GAP sporozoites (2.5 × 10^4^), α-OX40 treatment and challenge with wild type (PyWT) sporozoites (3 × 10^3^). Challenged mice were monitored for blood-stage infections from day 18 onwards to determine the prepatent period. **(C)** The Kaplan-Meier curves illustrate the prepatent period (day at which a parasitemia of 0.5–2% is observed). Data show representative from 2 independent experiments with (i) 5 and (ii) 10 mice per group: Naïve vs. GAP not significant (n.s.); GAP + α-OX40 vs. GAP; GAP + α-OX40 vs. Naïve, respectively in both experiments.

To determine whether OX40 is expressed on activated T cells in mice immunized with 2.5 × 10^4^ *P. yoelii fabb/f* GAP sporozoites, we determined the OX40 cell surface expression at day 3 post-immunization. OX40 expression was clearly detected on activated (CD44^hi^) CD4^+^ and CD8^+^ T cells in the liver (Figure 2A). In the spleen, the expression of OX40 on activated CD4^+^ T cells was also observed, albeit at lower levels compared to activated liver CD4^+^ T cells whereas OX40 expression on activated splenic CD8^+^ T cells was not detected (Figure 2A). These data show that GAP immunization is associated with the upregulation of co-stimulatory OX40 receptor on CD4^+^ and CD8^+^ T cells. Therefore to examine if α-OX40 treatment enhances protective immune responses after GAP vaccination, we treated mice with α-OX40 1 day after they were immunized with a single dose of 2.5 × 10^4^ GAP sporozoites as described above (Figure 2B). As expression of OX40 is upregulated after antigen recognition, we scheduled α-OX40 administration 1 day after the immunization (Aspeslagh et al., 2016). Immunized mice were injected intraperitoneally with 200 μg α-OX40 in 200 μl PBS by IP injection. In two experiments we observed an increase in protective immunity in GAP-immunized plus α-OX40 mice (Figure 2C). In the control groups of mice, naïve and GAP-immunized but not OX40 treated, none of the mice were protected against PyWT sporozoites challenge and all mice became patent at day 5 or 6 in two experiments. In contrast, in the two groups of GAP-immunized plus α-OX40 treated mice, a total of 4 out of 15 (26.7%) mice were completely protected and in 9 of the remaining 11 mice (60%) PyWT parasites emerged in the blood 1 day later than GAP-immunized mice and naive mice (Figure 2C). The vaccination of GAP with α-OX40 induced a significant increase in protection compared to immunization with only GAP parasites in both independent experiments (^*^p = 0.011 and ^**^p = 0.0017).

### α-OX40 treatment after a single gap immunization results in an increase of effector (CD44^hi^ CD11a^hi^) CD4^+^ T cells in both liver and spleen

**Figure 3:**
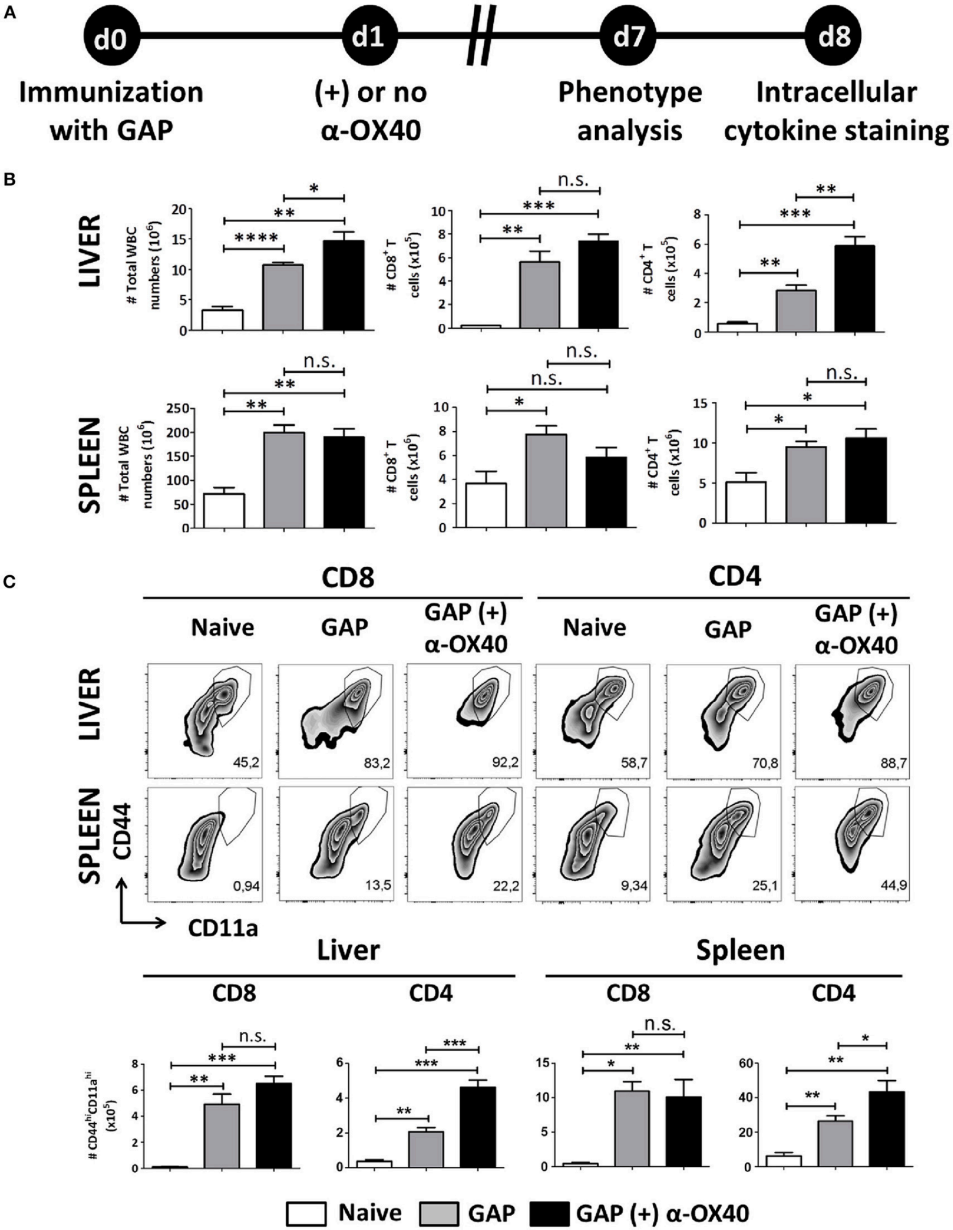
α-OX40 treatment after a single GAP immunization results in an increase of effector (CD44^hi^CD11a^hi^) CD4^+^ T cells in both liver and spleen. **(A)** The time line showing immunization of 2 groups of BALB/c mice with GAP sporozoites (2.5 × 10^4^) that were treated or not treated with α-OX40 one day after immunization. T cells were collected from the liver and spleen at day 7 and analyzed for phenotype analysis at day 7 or for cytokine expression at day 8 after *in vitro* re-stimulation with whole sporozoites. **(B)** The total number of WBC, CD8^+^ and CD4^+^ T cells in liver and spleen of different groups of mice. Significant differences in total WBC (*p = 0.03) and CD4^+^ T cells (**p = 0.0023) were observed between the livers of α-OX40 treated and non-treated mice. Representative data is shown from 2 independent experiments with 6 mice per group. **(C)** The upper panel shows the percentages of (CD44^hi^CD11a^hi^) T cells of total CD8^+^ and CD4^+^ T cells in liver and spleen in the different groups of mice. The lower panel shows the total number of (CD44^hi^CD11a^hi^) CD8^+^ and (CD44^hi^CD11a^hi^) CD4^+^ T cells in liver and spleen. A significant increase of (CD44^hi^CD11a^hi^) CD4^+^ cells was observed in both liver and spleen of mice immunized with GAP plus α-OX40 compared to only GAP-immunized mice (***p = 0.0004 and *p = 0.044, respectively). Representative data is shown from 2 independent experiments with 6 mice per group. Significant difference by unpaired *t-*test is indicated by not significant (n.s.) and significant; *p < 0.05 or **p < 0.01, ***p < 0.001, ****p < 0.0001.

One week after immunization, organs and blood were collected from mice that were immunized with a single dose of 2.5 × 10^4^ GAP sporozoites either with or without α-OX40 treatment (Figure 3A). In all immunized mice we observed a significant and strong increase in total white blood cells (WBCs) and CD4^+^/CD8^+^ T cells compared to the naïve control mice. Strikingly, we observed only in the GAP-immunized plus α-OX40 treated mice a significant increase (^**^p = 0.0023) in CD4^+^ T cell numbers in the liver compared to GAP-immunized mice (Figure 3B). We analyzed the phenotype of the antigen-experienced effector T cells using CD44 and CD11a as markers (Rai et al., 2009; Schmidt et al., 2010; Cooney et al., 2013). When we compared effector (CD44^hi^CD11a^hi^) CD8^+^ and CD4^+^ T cells we found that in both the spleen and the liver of GAP-immunized plus α-OX40 treated mice the number of (CD44^hi^CD11a^hi^) CD4^+^ T cells were significantly increased (^*^p = 0.044 and ^***^p = 0.0004; respectively), compared to GAP-immunized mice. No significant differences were observed in (CD44^hi^CD11a^hi^) CD8^+^ T cells in α-OX40 treated or untreated GAP-immunized mice, either in the liver or spleen (Figure 3C). Combined these results show that the administration of α-OX40 after a priming GAP immunization enhances the number of antigen-experienced effector CD4^+^ T cells in both the liver and spleen.

### Increased effector T cell formation by α-OX40 treatment after prime-boost gap immunization

**Figure 4:**
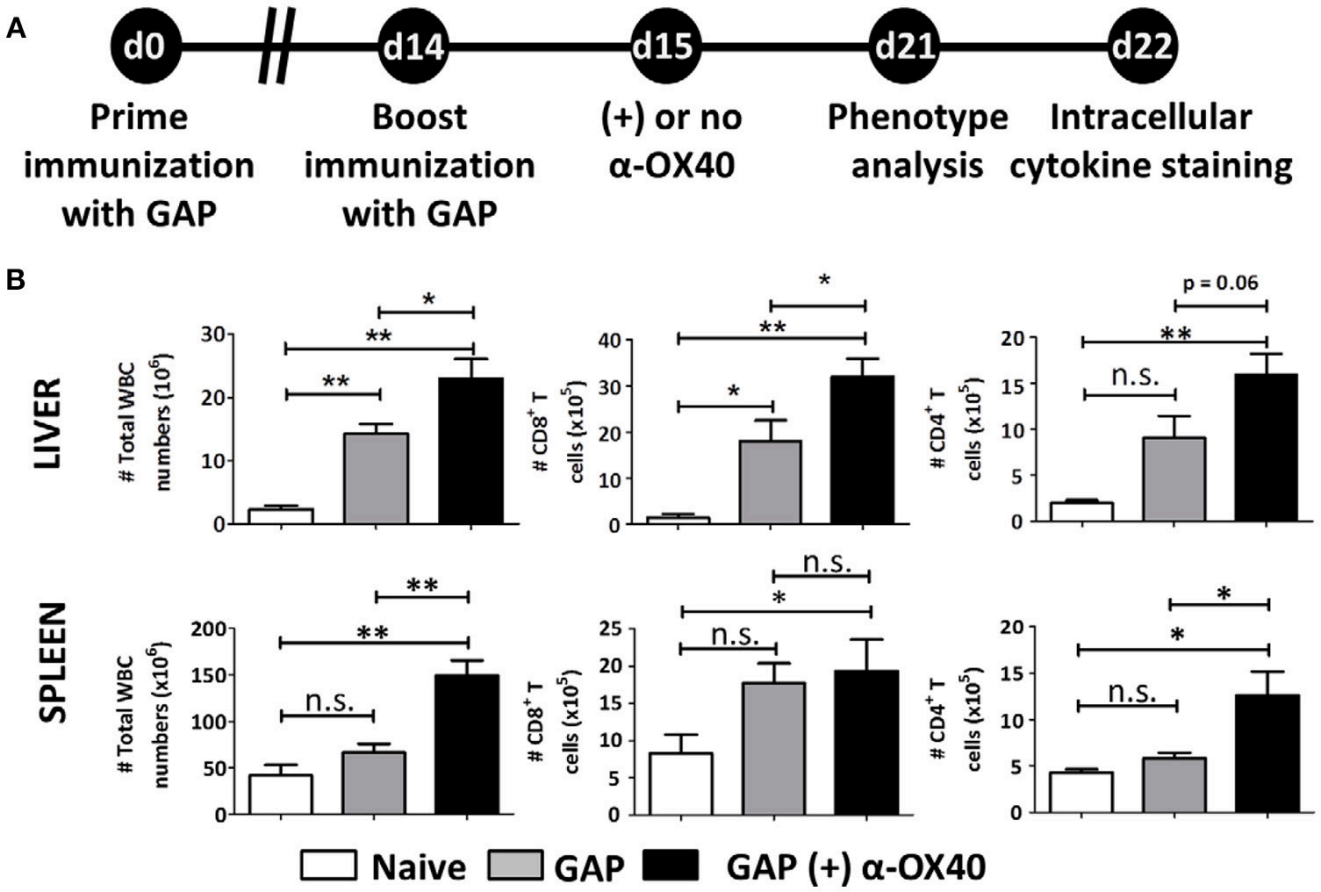
Prime-boost GAP immunization plus α-OX40 during the boost provokes the expansion of total WBC, CD8^+^ and CD4^+^ T cell numbers in the liver and spleen. **(A)** Time line showing immunization of 2 groups of BALB/c mice with GAP sporozoites (2.5 × 10^4^). Both groups received a prime (day 0) and boost (day 14) immunization, and were either treated or not treated with α-OX40 one day after the boost immunization. T cells were collected from the liver and spleen at day 7 for phenotype analysis at day 7 or for cytokine expression at day 8 after *in vitro* re-stimulation with whole sporozoites. **(B)** The total number of WBC, CD8^+^ and CD4^+^ T cells in liver and spleen of different groups of mice. Significant differences were observed between total WBC (*p = 0.035 and **p = 0.001) and CD4^+^ T cells (*p = 0.0625 and *p = 0.019) collected from the liver and spleen of treated and untreated mice. In addition, a significant difference in total CD8^+^ T cells (*p = 0.019) was observed between livers of treated and untreated mice. Representative data is shown from 2 independent experiments with 6 mice per group. Significant difference by unpaired *t-*test is indicated as not significant (n.s.) or significant; *p < 0.05 or **p < 0.01.

**Figure 5:**
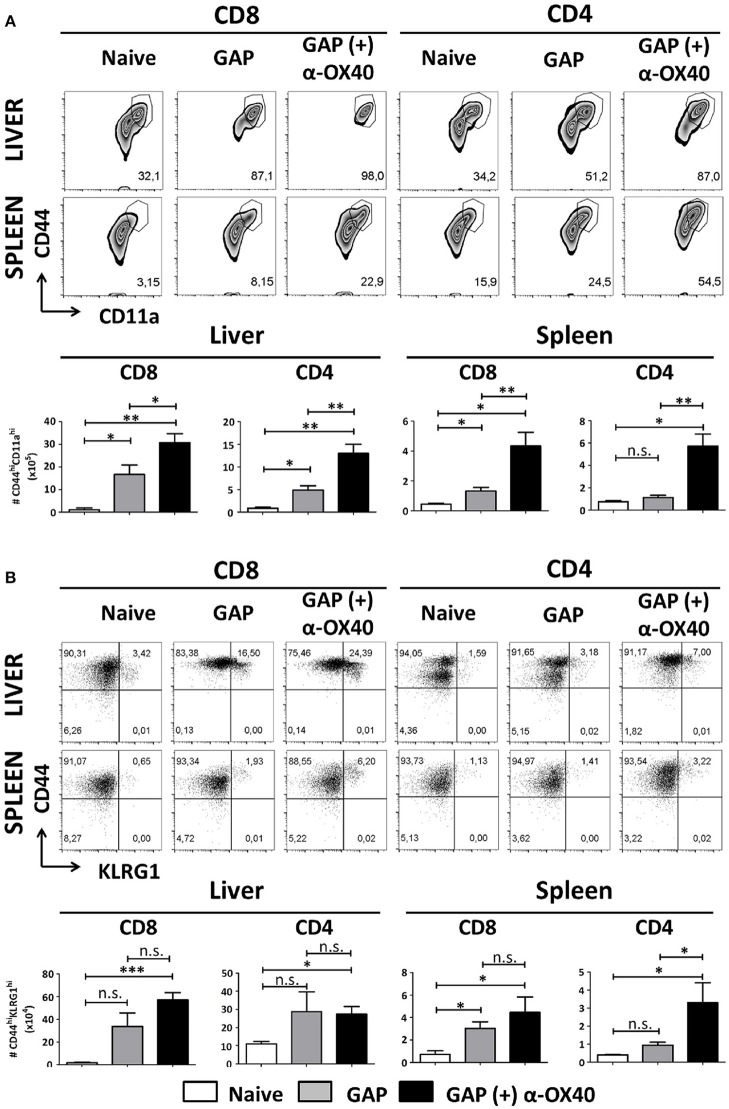
Increased effector T cell formation by α-OX40 treatment after prime-boost GAP immunization. **(A)** See Figure 4 for the time line of immunization and collection of T cells. The upper panel shows the percentages of (CD44^hi^CD11a^hi^) CD8^+^ and CD4^+^ T cells in liver and spleen. The lower panel shows the total number of (CD44^hi^CD11a^hi^) CD8^+^ and (CD44^hi^CD11a^hi^) CD4^+^ T cells in liver and spleen. A significant increase of (CD44^hi^CD11a^hi^) CD8^+^ cells (*p = 0.0357 and **p = 0.0073) and (CD44^hi^CD11a^hi^) CD4^+^ (**p = 0.0045 and **p = 0.0014) was observed in both liver and spleen, in mice immunized with GAP plus α-OX40 compared to only GAP-immunized mice. Representative data is shown from 2 independent experiments with 6 mice per group. Significant difference by unpaired *t-*test is indicated by not significant (n.s.) and significant; *p < 0.05 or **p < 0.01, ***p < 0.001. **(B)** The upper panel shows percentages of (CD44^hi^KLRG1^hi^) CD8^+^ and CD4^+^ T cells in liver and spleen in the different groups of mice. The lower panel shows the total number of (CD44^hi^KLRG1^hi^) CD8^+^ and (CD44^hi^KLRG1^hi^) CD4^+^ T cells. A significant increase of (CD44^hi^KLRG1^hi^) CD4^+^ T cells was observed in spleens of mice immunized with GAP plus α-OX40 compared to only GAP-immunized mice (*p = 0.043, respectively). Representative data is shown from 2 independent experiments with 6 mice per group. Significant difference by unpaired *t-*test is indicated by not significant (n.s.) and significant; *p < 0.05 or **p < 0.01, ***p < 0.001.

In order to examine the effect of α-OX40 treatment on both the formation and recall of the adaptive immune response after GAP vaccination, we adopted a prime-boost immunization strategy. Specifically, we immunized mice initially with a 2.5 × 10^4^ GAP sporozoites followed 2 weeks later by a boost with 2.5 × 10^4^ GAP sporozoites (Figure 4). This GAP immunization schedule provides 90–100% sterile protective immunity in BALB/c mice (Butler et al., 2011). α-OX40 treatment, as described above, was performed 1 day after the boost immunization as described for a vaccination protocol against mouse cytomegalovirus infection (Panagioti et al., 2017), and organs and blood collected 1 week after the boost immunization. In GAP-immunized plus α-OX40 treated mice we observed a strong significant increase in total WBCs in both the spleen and the liver (^**^p = 0.001) and liver (^*^p = 0.035) compared to GAP-immunized mice (Figure 4). We also observed an increase in total CD4^+^ and CD8^+^ T cells in GAP-immunized plus α-OX40 mice in the liver (*p* = 0.0625, ^*^p = 0.043 respectively). In the spleen we only observed a significant increase in total CD4^+^ T cells (^*^p = 0.019) but not in the CD8^+^ T cells in GAP-immunized plus α-OX40 treated mice compared to GAP-immunized mice. In both the spleen and the liver of GAP-immunized plus α-OX40 treated mice the number of (CD44^hi^CD11a^hi^) CD4^+^ (^**^p = 0.0014 and ^**^p = 0.0045; respectively) and CD8^+^ (^**^p = 0.0073 and ^*^p = 0.0357; respectively) T cells were significantly increased compared to GAP-immunized mice (Figure 5A). Also, when we compared activated effector-type (CD44^hi^KLRG1^hi^) CD4^+^ and CD8^+^ T cells, we found that in the spleens of GAP-immunized plus α-OX40 mice the number of (CD44^hi^KLRG1^hi^) CD4^+^ T cells were significantly increased (^*^p = 0.043) compared to GAP-immunized mice (Figure 5B). Combined these results suggest that enforced OX40 stimulation after a prime-boost immunization does not only impact the expansion of antigen-experienced effector CD4^+^ T cells, as was shown after a single immunization, but also expands the pool of antigen-experienced effector CD8^+^ T cells.

### α-OX40 treatment increases IFN-γ and TNF producing CD4^+^ T cells in both liver and spleen and increases the amount of sporozoite-specific antibodies after prime-boost gap immunization

**Figure 6:**
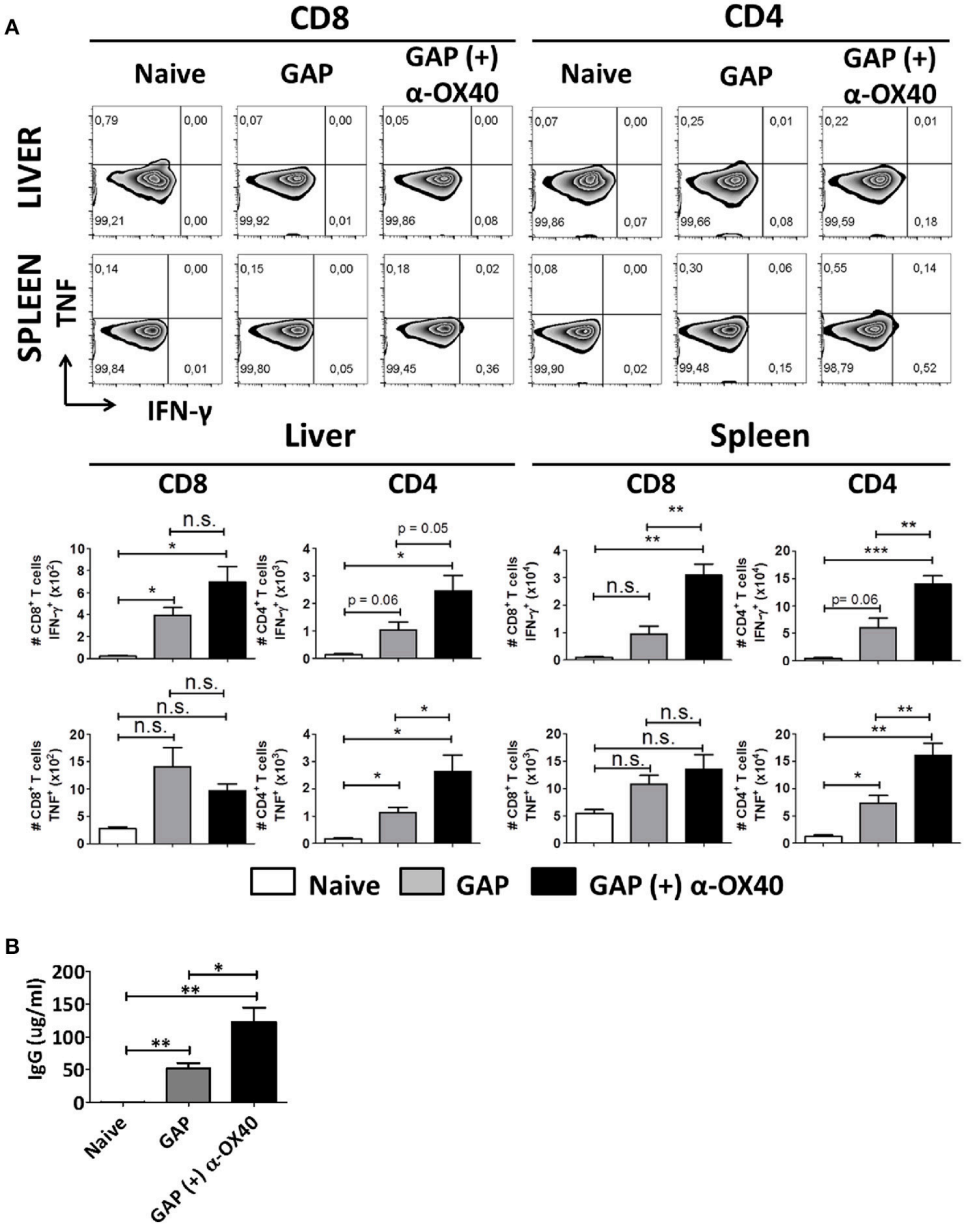
α-OX40 treatment increases IFN-γ and TNF producing CD4^+^ T cells in both liver and spleen and increases the amount of sporozoite-specific antibodies after prime-boost GAP immunization. **(A)** See Figure 4 for the time line of immunization and collection of T cells. The upper panel shows percentage of IFN-γ and TNF cytokine producer CD8^+^ and CD4^+^ T cells in liver and spleen after *in vitro* sporozoite re-stimulation. The second panel shown the total number of IFN-γ and TNF cytokine producer CD8^+^ and CD4^+^ T cells in liver and spleen. Significant differences in both IFN-γ cytokine producer CD8^+^ and CD4^+^ T cells (**p = 0.0018 and **p = 0.0065, respectively) were observed between spleens of α-OX40 treated and untreated mice. In addition, an increase in IFN-γ cytokine producer CD4^+^ T cells (*p* = 0.0506) was observed in livers of treated mice compared to livers of untreated mice. Further, a significant differences of TNF producing CD4^+^ T cells in both liver and spleen (*p = 0.0398 and **p = 0.0068, respectively) were observed between α-OX40 treated and untreated mice. Representative data is shown from 2 independent experiments with 6 mice per group. Significant difference by unpaired *t-*test is indicated by not significant (n.s.) and significant; *p < 0.05 or **p < 0.01, ***p < 0.001. **(B)** Quantification by ELISA of *P. yoelii* WT sporozoite-specific IgG obtained from naïve and prime-boost immunized mice with *P. yoelii* GAP (with or without α-OX40 treatment). The concentration of the total IgG in the ELISA was quantified using the values for each sample based on the standard curve obtained with defined concentrations of polyclonal antibodies against PyCSP (Bioss Antibodies Inc., USA).

In order to study the impact of the α-OX40 treatment on the cytokine production of the CD4^+^ and CD8^+^ T cells after prime-boost immunization, we performed intracellular staining for IFN-γ and TNF of hepatic leucocytes and splenocytes isolated 7 days after the final immunization. Before staining cells were stimulated *in vitro* for a period of 24 h with sporozoites. It has been reported that treatment with anti-ARTC2 antibodies can improve T cell survival and recovery after *in vitro* stimulation consequently mice were treated with ARTC2 nanobodies 30 min before collection of the organs (Rissiek et al., 2014). We observed a significant increase in IFN-γ producing CD4^+^ (^**^p = 0.0065) and CD8^+^ (^**^p = 0.0018) T cells in the spleens of GAP-immunized plus α-OX40 treated mice compared to GAP-immunized mice. In the liver of GAP-immunized plus α-OX40 treated mice, there was an increase of IFN-γ producing CD4^+^ T cells (*p* = 0.0506) but not of CD8^+^ T cells. Further, we observed a significant increase in TNF producing CD4^+^ T cells in both liver (^*^p = 0.0398) and spleen (^**^p = 0.0068) of GAP-immunized plus α-OX40 treated mice compared to GAP-immunized mice but TNF production in CD8^+^ T cells was not significantly different in either the liver or spleen (Figure 6A). Taken together, these results show that α-OX40 treatment after a prime-boost GAP immunization elicits a significant increase in IFN-γ and TNF producing CD4^+^ T cells in both liver and spleen of GAP-immunized plus α-OX40 treated mice compared to GAP-immunized mice.

In addition to collecting organs at day 7 after the final immunization, we also collected serum from these mice to perform ELISA analysis with *P. yoelii* sporozoite lysate to quantify parasite-specific IgG responses. This analysis revealed that mice immunized with GAP sporozoites generate sporozoite-specific antibody responses and that there is a significant (^*^p = 0.02, Student's *t*-test) increase in the total IgG produced in mice immunized with GAP plus α-OX40 treatment compared to mice immunized with only GAP (Figure 6B).

## Discussion

Vaccination with live attenuated sporozoites can induce protective immunity in humans but induction of sterile protection requires immunization with multiple doses and each dose consisting of relatively high numbers of sporozoites (Hoffman et al., 2015; Hollingdale and Sedegah, 2017). Enhancing the immunogenicity of whole sporozoite (wsp) vaccines, for example by adding adjuvants, can be used to both reduce the number of sporozoites per dose and the number of vaccine doses, as well as directing the adaptive immune response. We show in this study that treatment of mice with an agonistic antibody against the T cell costimulatory molecule OX40, a member of the tumor necrosis factor receptor (TNFR) superfamily (AlShamkhani et al., 1996), enhances protective immunity after immunization with GAP sporozoites.

Previously it has been shown that targeting OX40 increases the magnitude of T cell responses and improves T cell functionality (Sugamura et al., 2004; Croft, 2010). OX40 is transiently expressed on T cells following cognate interactions between T cell receptors (TCRs) and antigen-major histocompatibility (MHC) complexes on antigen presenting cells (APCs) (Croft, 2010). While OX40 is expressed on both activated CD4^+^ and CD8^+^ T cells, OX40 expression on CD4^+^ T cells is greater than CD8^+^ T cells and consequently α-OX40 treatment is expected to exert its greatest effect on CD4^+^ T cells (Baum et al., 1994; AlShamkhani et al., 1996; Croft, 2010). OX40 signaling promotes T cell proliferation and survival, influences CD4^+^ T cell differentiation into T helper subsets (Walker et al., 1999; Murata et al., 2000; Soroosh et al., 2007; Song et al., 2008) and is reported to reverse CD4^+^ T cell hypo-responsiveness (Bansal-Pakala et al., 2001). While it has been previously described that OX40 is expressed on activated human and rodent CD4 T cells after a malaria blood stage infection (Zander et al., 2015; Goncalves-Lopes et al., 2016) no data had been reported on the expression of OX40 on T cells after a sporozoite/liver stage *Plasmodium* infection/immunization. We demonstrate in this study that after GAP-sporozoite immunization OX40 expression was observed on activated (CD44^hi^) CD4^+^ and CD8^+^ T cells in the liver. Similarly OX40 expression was upregulated on activated CD4^+^ T cells in the spleen but not observed on activated CD8^+^ T cells. We therefore hypothesized that therapeutic ligation of OX40 during immunization with attenuated *Plasmodium* sporozoites would increase parasite-specific CD4^+^ and CD8^+^ T cell activity, limit the degree of T cell exhaustion and improve T cell effector-memory formation, all resulting in increased clearance of PyWT sporozoites/liver stages.

To analyze the effect of adjuvants on wsp vaccination approaches we first developed a model with a sub-saturating immunization regiment, which we could use to measure enhancement of protective immunity through the application of adjuvants. In this study we demonstrate that a single immunization with 2.5 and 5 × 10^4^ sporozoites induces partial protection as determined by an absence of sterile protection after PyWT sporozoites challenge but a 1 day delay in the emergence of parasites in the blood (prepatent period). A 1 day delay in prepatent period has been correlated with a 10 × reduction in parasites released from the liver (Janse et al., 2006), indicating a 10 × increase in protective immunity compared to unimmunized mice.

In cytomegalovirus (CMV) vaccination studies it was found that the increase in vaccine potency can be achieved by α-OX40 treatment through the expansion of both antigen-specific CD4^+^ and CD8^+^ T cells (Panagioti et al., 2017). A marked upregulation of OX40 is observed on *Plasmodium* specific CD4^+^ T cells that are generated in both human and rodent malaria blood stage infections and, in rodent studies, α-OX40 treatment was shown to increase parasite-specific memory CD4^+^ T cells resulting in a reduced blood-stage infection (Zander et al., 2015, 2017; Goncalves-Lopes et al., 2016). However, prior to this study the effects of OX40 treatment on immune responses induced by wsp vaccination have not been described.

Our analyzes of T cell responses in mice immunized with a single dose of GAP parasites, showed an increase in total WBC numbers in the livers and an increase in CD4^+^ effector (CD44^hi^CD11a^hi^) T cells in both liver and spleen of α-OX40 treated mice compared to untreated mice. It has been reported by Cooney et al. that T cells with a CD44^hi^CD11a^hi^ phenotype are indicative of antigen-experienced effector cells in GAP-immunized BALB/c mice (Cooney et al., 2013). While protective immunity after wsp immunization is thought to largely dependent on the killing infected hepatocytes by CD8^+^ T cells and IFN-γ (Tarun et al., 2007; Douradinha and Doolan, 2011; Khan et al., 2012), adoptive transfer of CD4^+^ T cells from GAP-immunized C57BL/6 mice was able to provide sterile protection to 50% of naïve animals against a WT infection, indicating an important role for CD4^+^ T cells in GAP induced immunity (Tarun et al., 2007). In addition, protective immunity induced by sporozoites of a *P. yoelii* GAP, similar to the one used in our study, was dependent not only on CD8^+^ T cells but also CD4^+^ T cells (Murray et al., 2015). Immune responses induced by *P. yoelii* GAPs that arrest late into liver development (Butler et al., 2011) are reported to involve both the cellular and humoral arm of the adaptive immune response. Indeed, Keitany *et al*. showed that functional antibodies are induced after immunization with *P. yoelii* GAPs, which can inhibit sporozoite invasion of liver cells and reduce intrahepatic parasite development (Vanderberg and Frevert, 2004; Keitany et al., 2014; Sack et al., 2014). Since enhancement of CD4^+^ T cell responses by OX40 stimulation may lead to an increase in humoral immunity we examined total IgG responses generated in mice after prime-boost GAP immunization, either with or without α-OX40 treatment. These studies revealed that anti-sporozoite antibodies were generated after GAP immunization and significantly more IgG was generated in mice immunized with GAP plus α-OX40 treatment compared to mice immunized with only GAP. This observation indicates that the increase in CD4^+^ T cells after α-OX40 treatment may be directly contributing to B cell maturation/activation.

We further examined the effect of α-OX40 treatment on adaptive immune responses by analyzing immune responses in mice that had received a boost immunization after the prime immunization. After this prime-boost strategy we observed an increase in total WBC numbers in livers and spleens of both GAP-immunized plus α-OX40 and GAP-only immunized mice. However, we observed a significant increase in effector (CD44^hi^CD11a^hi^) CD4^+^ and CD8^+^ T cells in liver and spleen of GAP-immunized plus α-OX40 treated mice compared to GAP-only immunized mice. This is in contrast to the single prime strategy where we only observed a significant in increase only in effector (CD44^hi^CD11a^hi^) splenic and liver CD4^+^ T cells in GAP-immunized plus α-OX40 treated mice. When we examined the activation phenotype (CD44^hi^KLRG1^hi^) of these T cells in liver and spleen, α-OX40 treatment significantly increased only the number of activated CD4^+^ T cells and only those present in the spleen. Additionally, we observed a significant increase in IFN-γ producing CD4^+^ and CD8^+^ T cells in the spleen but not in the liver (Murray et al., 2015). We also observed a significant increase in TNF producing CD4^+^ T cells, but not CD8^+^ T cells, in the liver and spleens of GAP-immunized plus α-OX40 treated mice. Therefore CD4^+^ T cells in the spleen may contribute to protective immunity either by enhancing humoral responses targeting sporozoites invasion (Sack et al., 2014) or by enhancing CD8^+^ T cell responses that target infected hepatocytes. Recently it was reported, in mice that liver resident CD8^+^ T cells induced by wsp vaccination may be primed in the spleen and their conversion occurring after reencountering parasite antigen in the liver (Fernandez-Ruiz et al., 2016). Our results indicate that the increased protective immunity observed in GAP-immunized plus α-OX40 treated mice acts primarily via enhanced CD4^+^ T cell responses in the spleen. It is known that CD4^+^ T cell help is necessary for an effective CD8^+^ T cell memory response against non-inflammatory antigens, such as tumor cells and certain pathogens that may not carry sufficient danger signals (Sun et al., 2004). Mice depleted of CD4^+^ T cells during immunization with sporozoites failed to exhibit a robust CD8^+^ T cell expansion and were not protected against challenge (Weiss et al., 1993; Overstreet et al., 2011). Murray *et al*. found that CD4^+^ T cell help was also necessary to induce protection after immunization with GAP sporozoites (Murray et al., 2015). OX40, in addition to being a costimulatory receptor that potentiates proliferation, survival, memory formation, and effector function of CD4^+^ and CD8^+^ T cells, can also overcome the suppressive activity of regulatory T cells (Tregs) (Croft et al., 2009). Overcoming immune suppression effects could also benefit the generation of protective immunity after wsp vaccination as it has been recently shown that wsp immunization, in particular after GAP administered via the skin, can induce regulatory responses (Haeberlein et al., 2017). Together, our results indicate that improving CD4^+^ T cell activation enhances protective immunity against malaria. Whether this CD4^+^ T cell stimulation acts primarily by improving humoral responses targeting sporozoites or by increasing CD8^+^ T cell responses against infected liver cells and how these responses may contribute to formation of immunological memory and duration of protection requires further investigation.

A limited number of other studies have been performed on the effect of adjuvants on protective immunity induced by wsp immunization. In particular the use of the glycolipid α-galactosylceramide (α-GalCer) (Gonzalez-Aseguinolaza et al., 2002) and its analog 7DW8-5 have been analyzed (Li et al., 2015). Co-administration of these molecules with sporozoites resulted in enhanced recruitment and activation/maturation of dendritic cells in lymph nodes draining the site of vaccine administration and thereby enhancing parasite-specific T cell immunogenicity. Although the possible use of certain adjuvants in human vaccination studies may be difficult due to costs, applicability or side-effects, these pre-clinical studies provide useful information of the largely unknown mechanisms underlying protective immunity. Although α-OX40 treatment is currently in clinical trials for cancer immunotherapy, the use of antibody-based α-OX40 treatment may, for vaccines for the developing world, be unrealistic as they are likely to be too expensive. Other (protein based) agents that can stimulate costimulatory responses, including agonists of OX40 are being developed as potential adjuvants in vaccine development. For example, combination therapy using the protein ligand of OX40, OX40L, fused to a cancer vaccine have been shown to reduce breast cancer metastasis, by enhancing antigen specific CD4^+^ and CD8^+^ T cell responses and inhibiting immunosuppressive Treg responses (Malamas et al., 2017). The co-administration of proteins like OX40L which are likely to be cheaper and easier to produce, may therefore be more practical and feasible approaches to pursue. In conclusion, this study demonstrates how specific immune response to vaccination coupled with activation of costimulatory molecules on the surface of T cells, can enhance protective immunity after wsp immunization and merits further investigation to see if such approaches not only increase the magnitude but also the breadth of an immune responses after vaccination.

## Author contributions

AO designed and performed most of the experiments and data analysis, and wrote the manuscript. BF-F performed the experiments and reviewed the manuscript. TI, EvdG, AR, and SC-M conducted experiments and assisted with flow cytometry. JR, AS, and CM-M generated the GAP mutant. CJ, RA, and SK designed and supervised the study and wrote the manuscript.

### Conflict of interest statement

The authors declare that the research was conducted in the absence of any commercial or financial relationships that could be construed as a potential conflict of interest.
