# Supplementary material for: OX40 Stimulation Enhances Protective Immune Responses Induced After Vaccination With Attenuated Malaria Parasites
Source: Front Cell Infect Microbiol. 2018 Jul 19;8:247. doi: 10.3389/fcimb.2018.00247 (PMC6060232; doi:10.3389/fcimb.2018.00247)
Supplement: Supplementary file 1 [file Table_1.DOCX]

| Parasites | Dose | Breakthrough/Infected animals^a^ | Prepatency (days) |
| --- | --- | --- | --- |
| PyWT | 3 × 10^3^ | 6/6 | 5 |
| GAP | 1 × 10^4^ | 0/4 | - |
|  | 2.5 × 10^4^ | 0/40 | - |
|  | 5 × 10^4^ | 0/4 | - |

^a^ Number of mice showing breakthrough infections of the total number of infected mice
