# Supplementary material for: OX40 Stimulation Enhances Protective Immune Responses Induced After Vaccination With Attenuated Malaria Parasites
Source: Front Cell Infect Microbiol. 2018 Jul 19;8:247. doi: 10.3389/fcimb.2018.00247 (PMC6060232; doi:10.3389/fcimb.2018.00247)
Supplement: Supplementary file 2 [file Data_Sheet_1.DOCX]

Supplementary Material

**OX40 stimulation enhances protective immune responses induced after vaccination with attenuated malaria parasites**

**Ahmad Syibli Othman^1,3^, Blandine M Franke-Fayard^1^, Takashi Imai^1^, Esmé T. I. van der Gracht^2^, Anke Redeker^2^, Ahmed M. Salman^1,4^, Catherin Marin-Mogollon^1^, Jai Ramesar^1^, Séverine Chevalley-Maurel^1^, Chris J. Janse^1^, Ramon Arens^2^*** **and Shahid M. Khan^1^***

Addresses:

^1^Leiden Malaria Research Group, Parasitology, Leiden University Medical Center (LUMC), Leiden, The Netherlands

^2^Department of Immunohematology and Blood Transfusion, Leiden University Medical Center (LUMC), Leiden, The Netherlands

^3^Faculty of Health Sciences, Universiti Sultan Zainal Abidin, Terengganu, Malaysia

^4^The Jenner Institute, University of Oxford, ORCRB, Roosevelt Drive, Oxford, United Kingdom

***Correspondence:**

Dr. Shahid M. Khan

[S.M.Khan@lumc.nl](mailto:S.M.Khan@lumc.nl)

Dr. Ramon Arens

[R.Arens@lumc.nl](mailto:R.Arens@lumc.nl)

**Supplementary FIGURE S1 |** Phenotype features of *P. yoelii* GAP (GAP) parasites compared to *P. yoelii* wildtype (PyWT) parasites.

**(A)** No significant differences in oocyst and sporozoite production in *Anopheles stephensi* mosquitoes between PyWT and GAP were observed. Oocysts and sporozoites were counted at day 8 and day 14 after the mosquito feeding, respectively.

**(B)** No significant differences in parasite liver load between mice infected with 1 × 10^4^ GAP sporozoites and mice infected with 1 × 10^4^ PyWT sporozoites IV at 44 h post infection, were observed. Parasite liver load in mice was determined by measuring *in vivo* luciferase activity and depicted as relative light units (RLU). The right panel shows representative images of real time in vivo imaging of luciferase expressing liver stage parasites in mice at 44 h after injection of PyWT and GAP sporozoites.

**Supplementary TABLE 1.** Breakthrough blood infections and prepatent period in mice after intravenous injection of different doses of PyWT and GAP sporozoites.
