# Supplementary figures and images for: OX40 Stimulation Enhances Protective Immune Responses Induced After Vaccination With Attenuated Malaria Parasites
Source: Front Cell Infect Microbiol. 2018 Jul 19;8:247. doi: 10.3389/fcimb.2018.00247 (PMC6060232; doi:10.3389/fcimb.2018.00247)

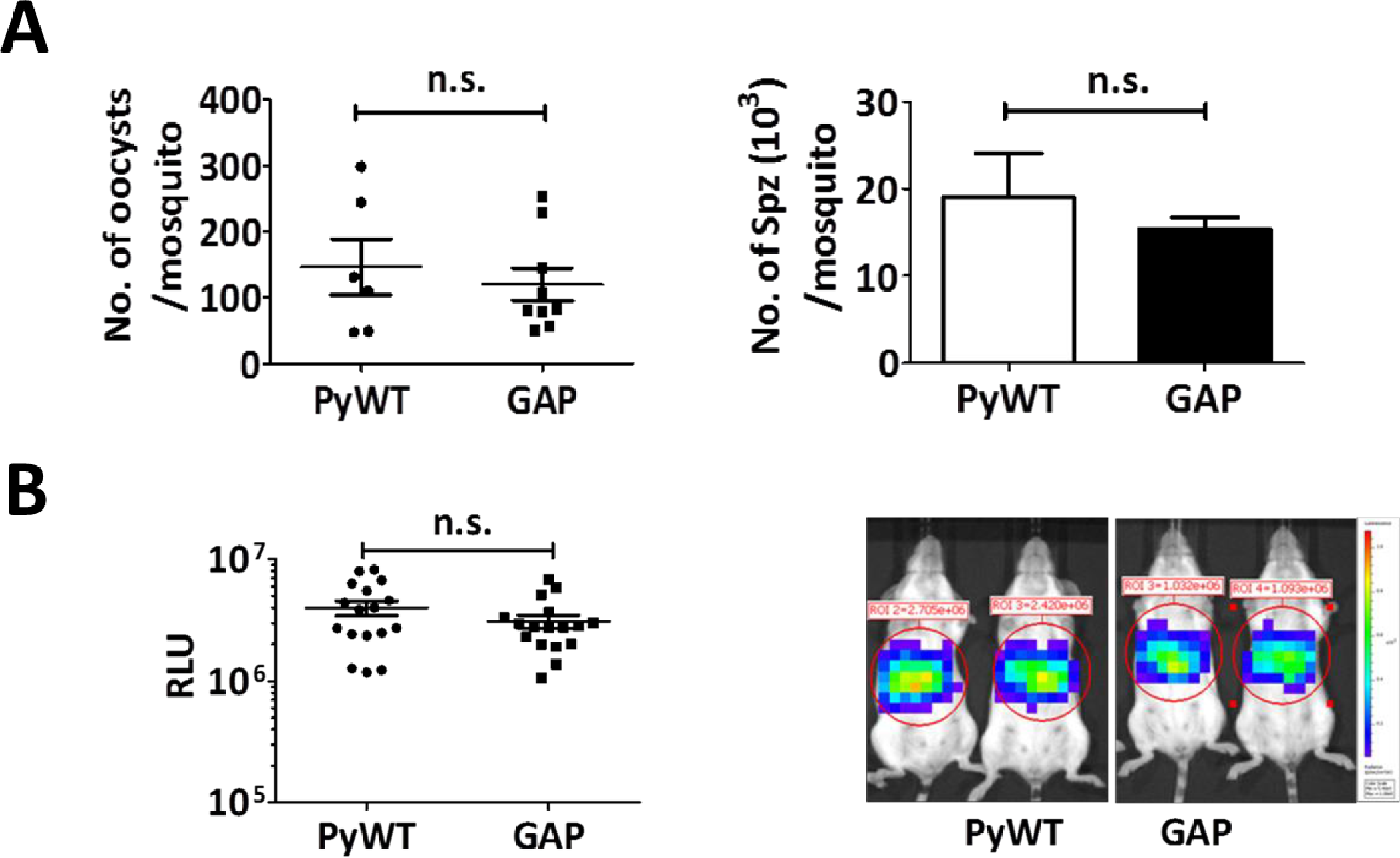

Supplement: Supplementary file 3 [file Image_1.TIF]
